# Supplementary figures and images for: Designer circRNAGFP reduces GFP-abundance in Arabidopsis protoplasts in a sequence-specific manner, independent of RNAi pathways
Source: Plant Cell Rep. 2025 May 22;44(6):128. doi: 10.1007/s00299-025-03512-y (PMC12098445; doi:10.1007/s00299-025-03512-y)

## Slide 1
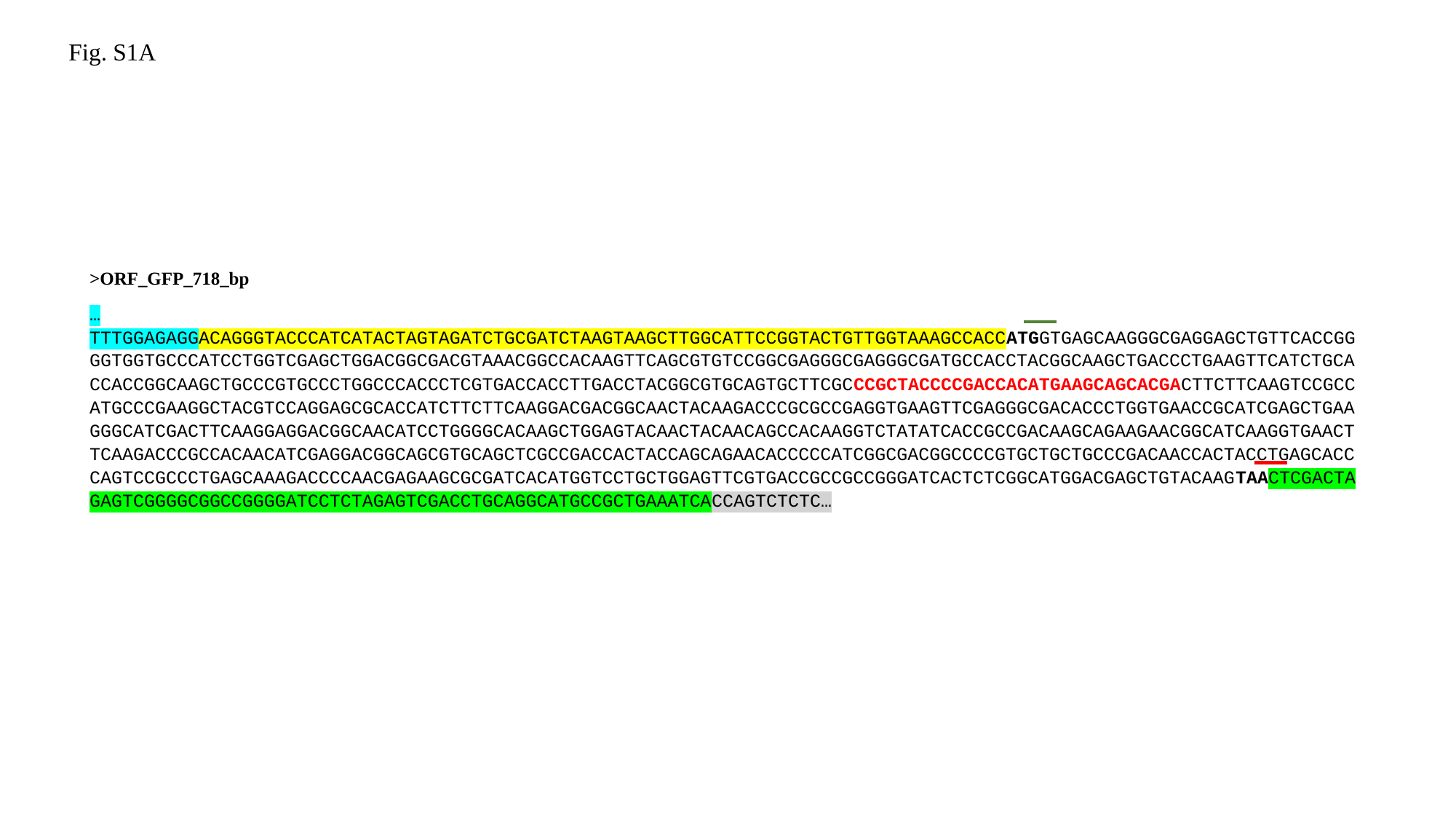

## Slide 2
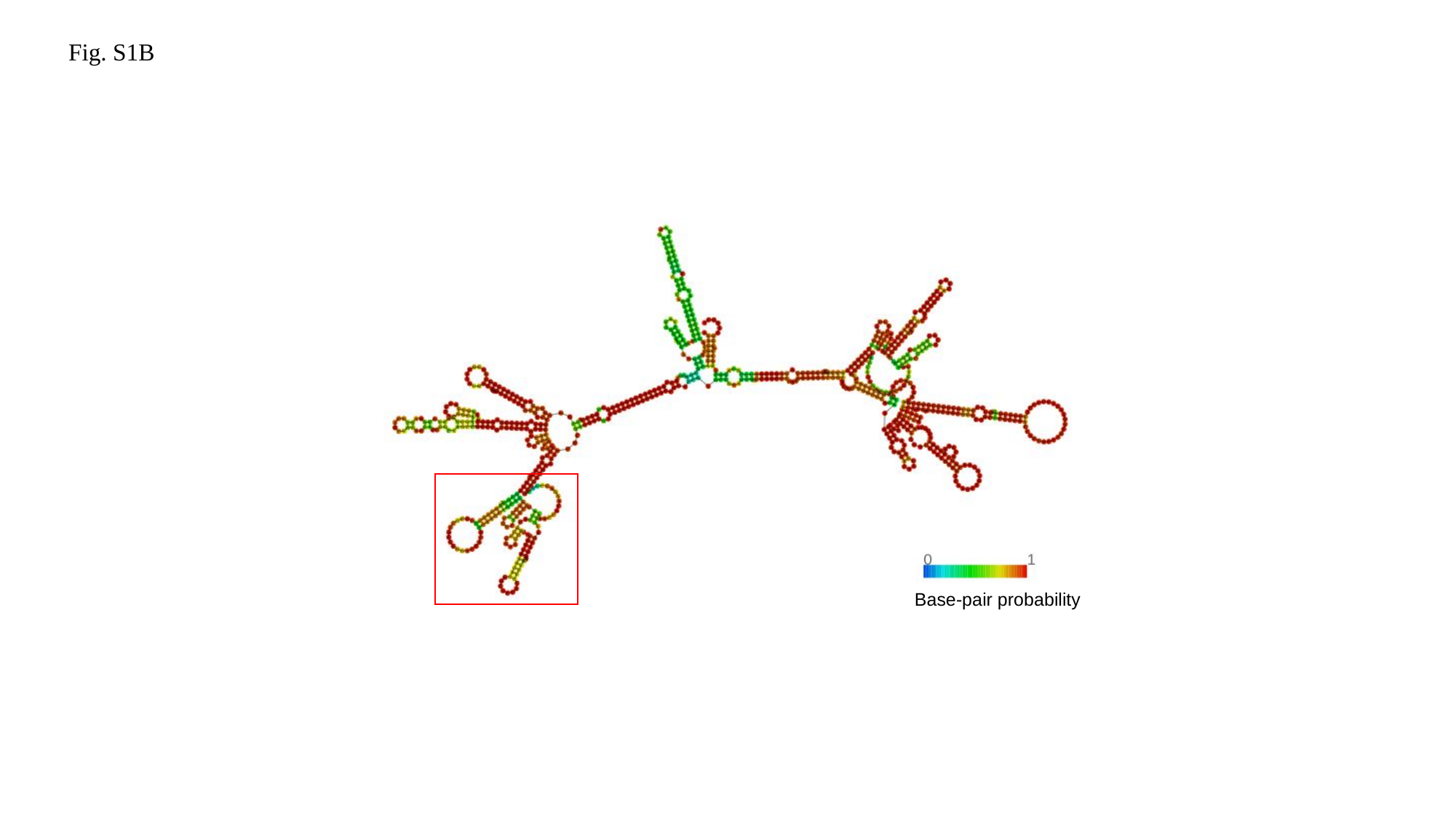

## Slide 3
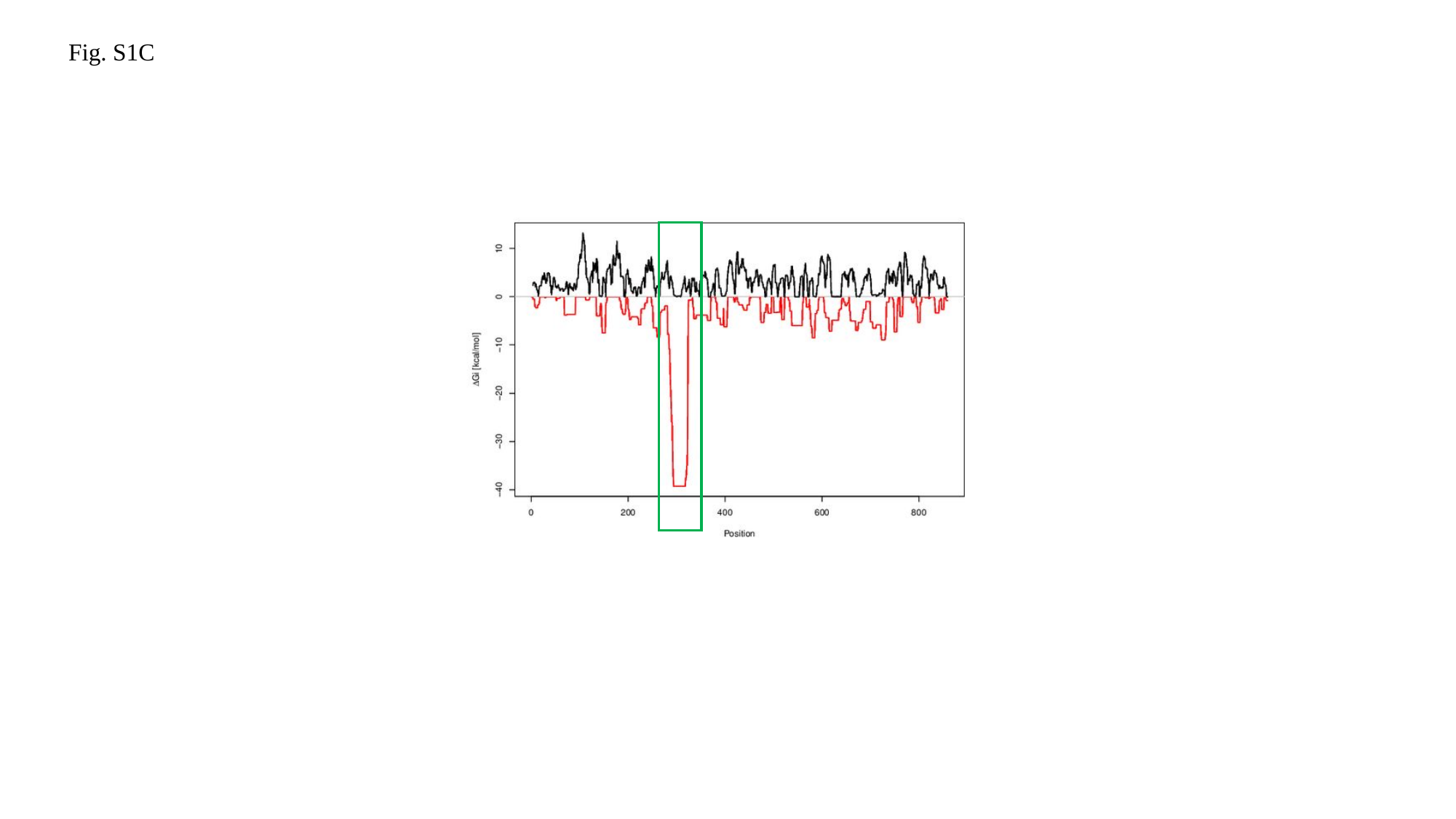

## Slide 4
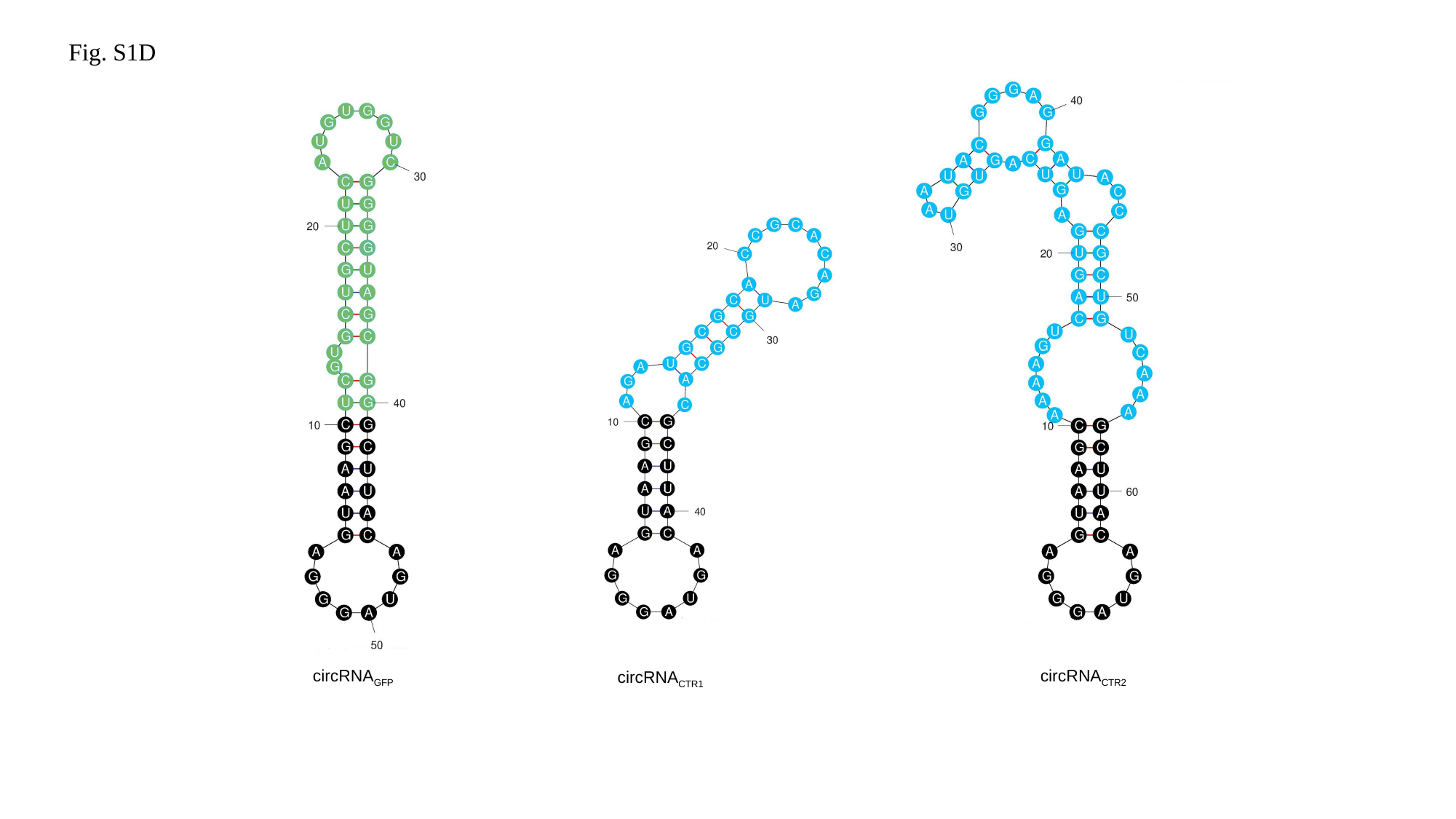

## Slide 5
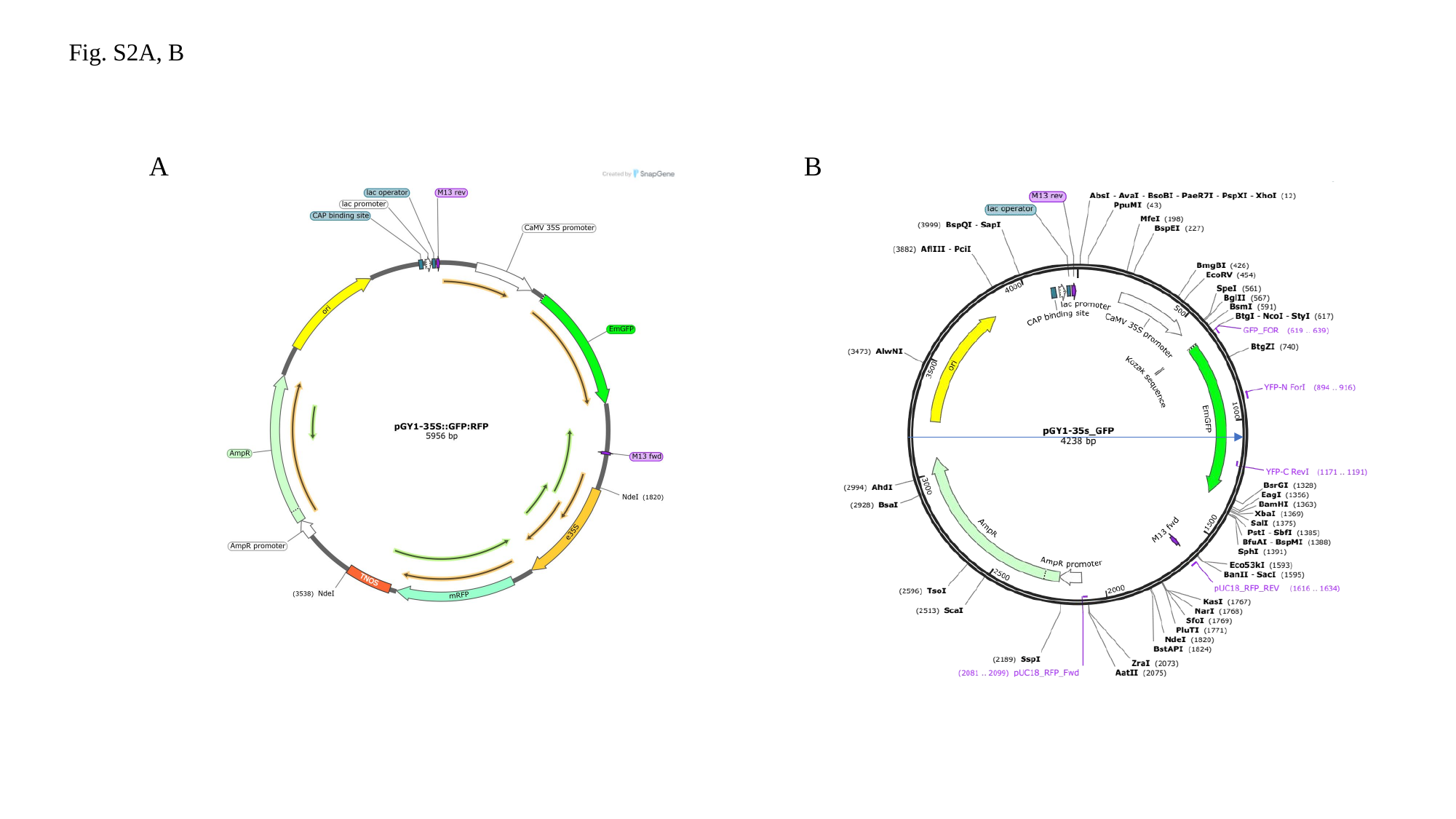

## Slide 6
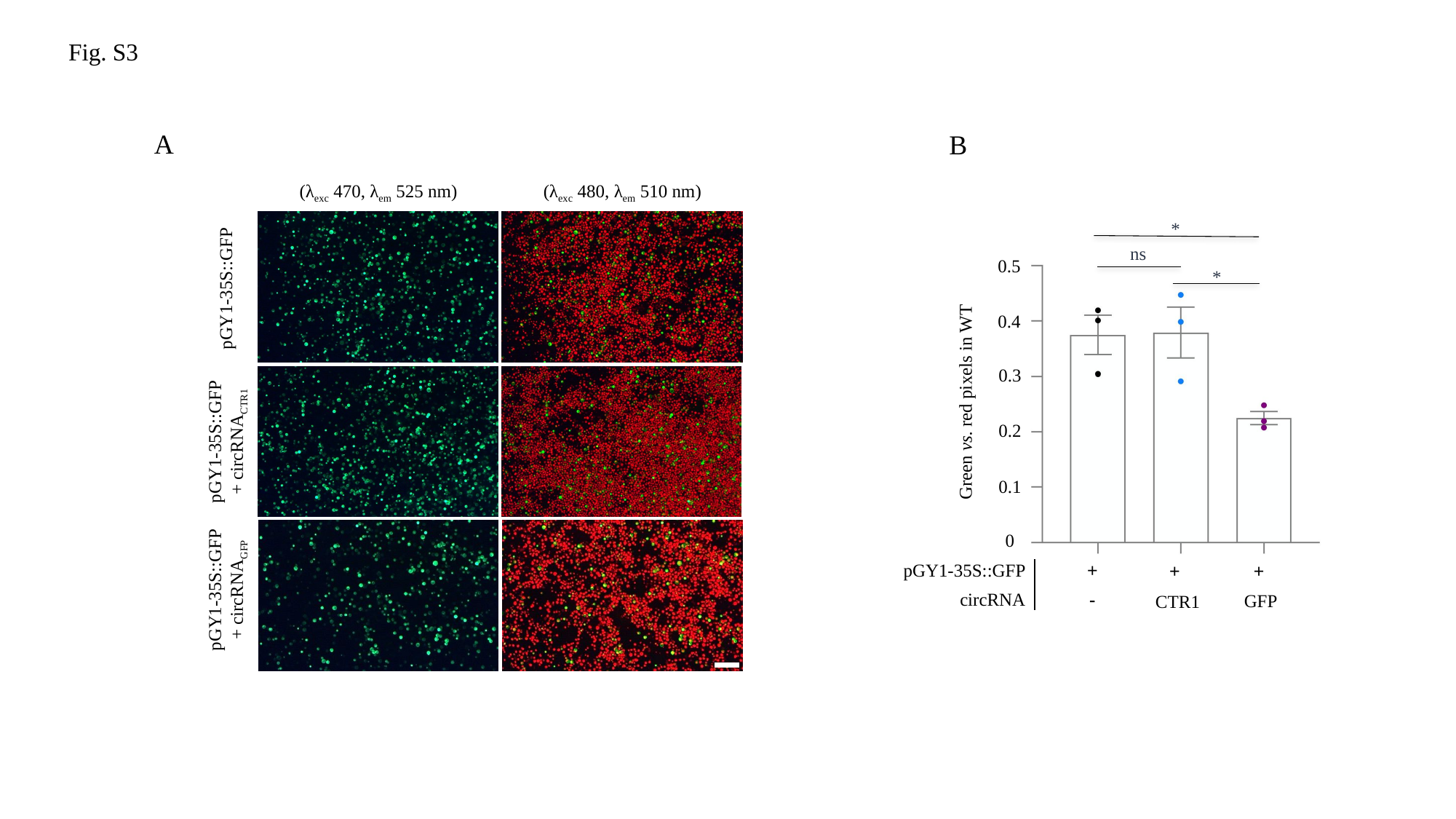

## Slide 7
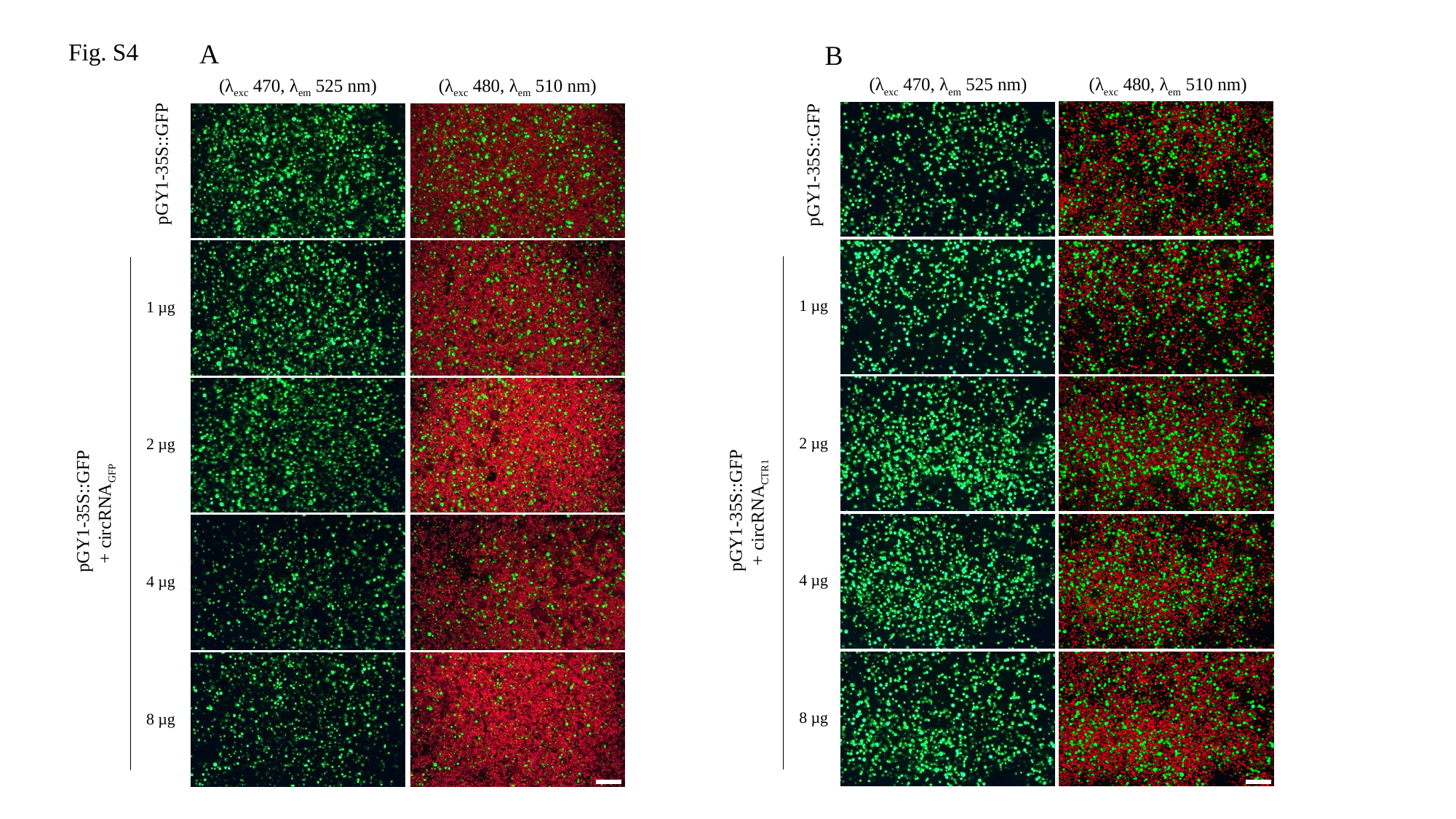

## Slide 8
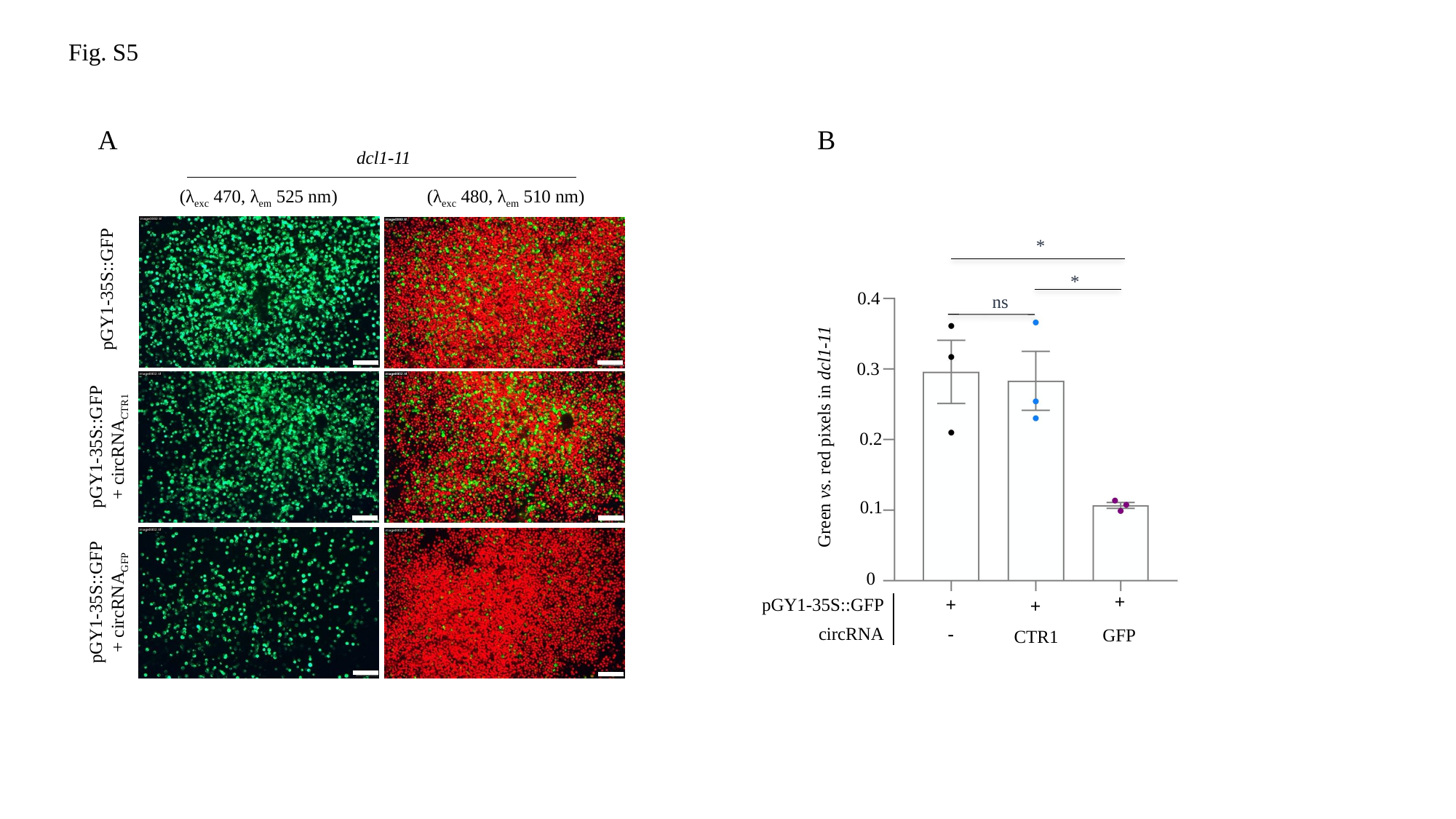

## Slide 9
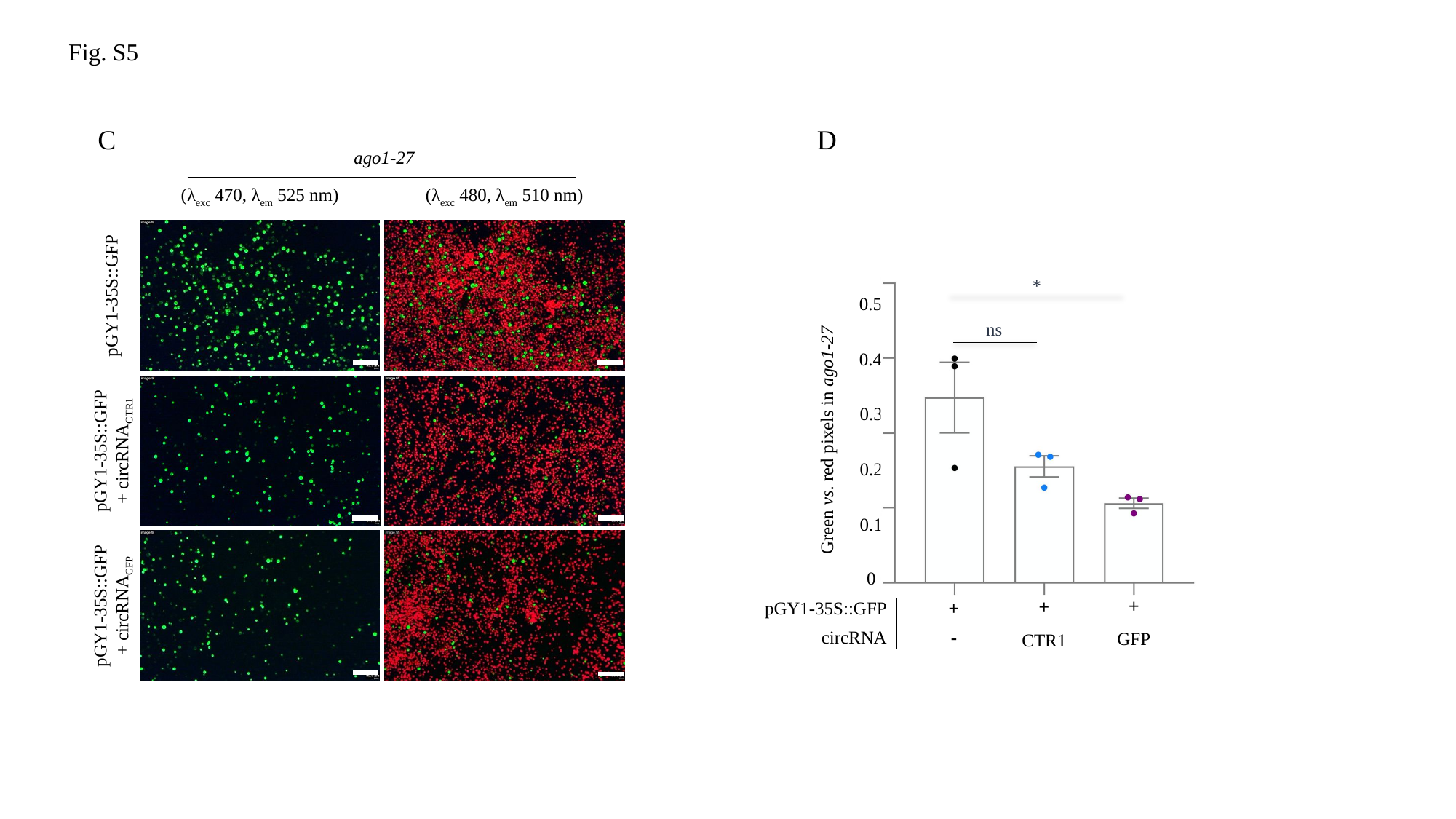

## Slide 10
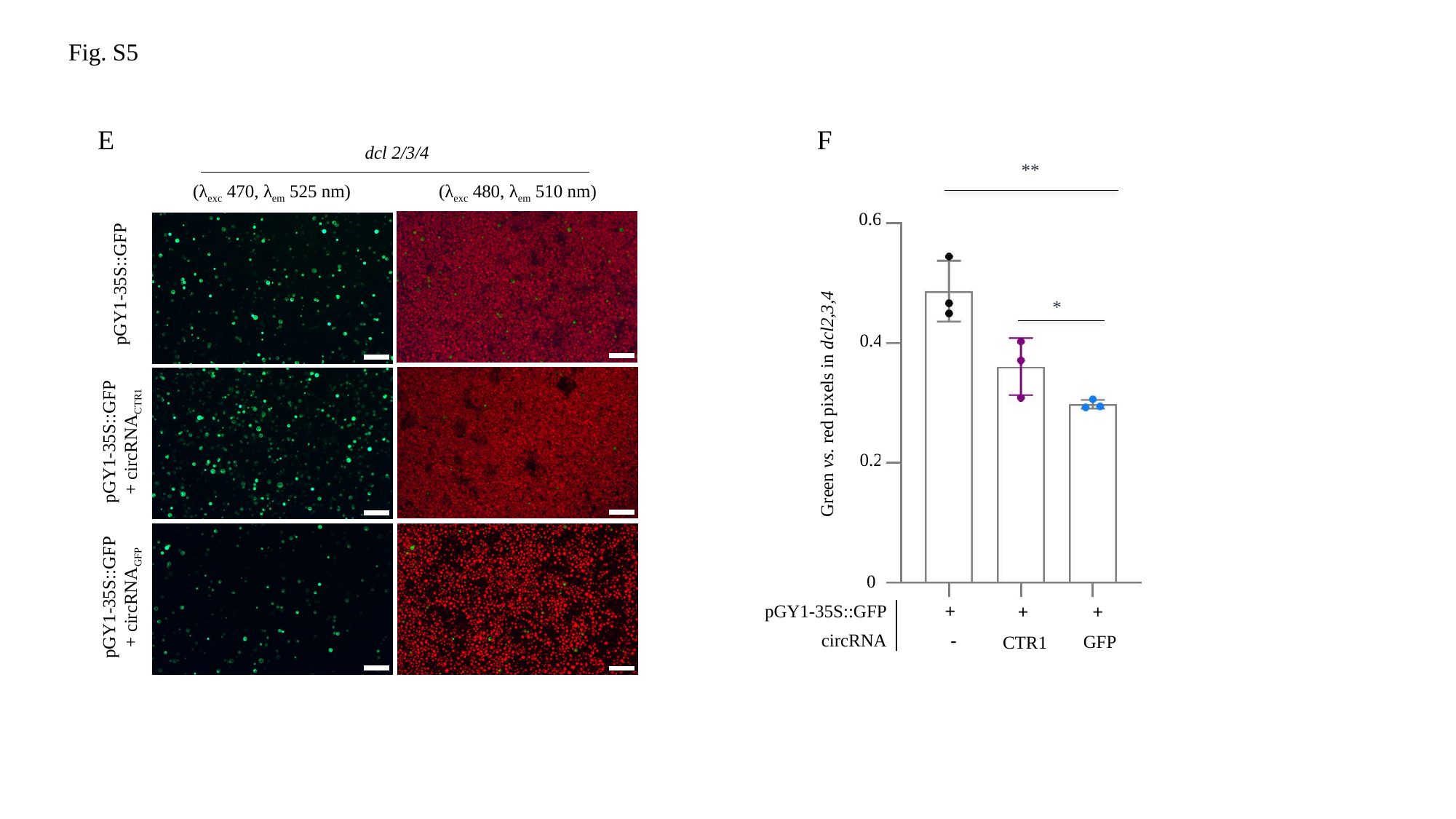

## Slide 11
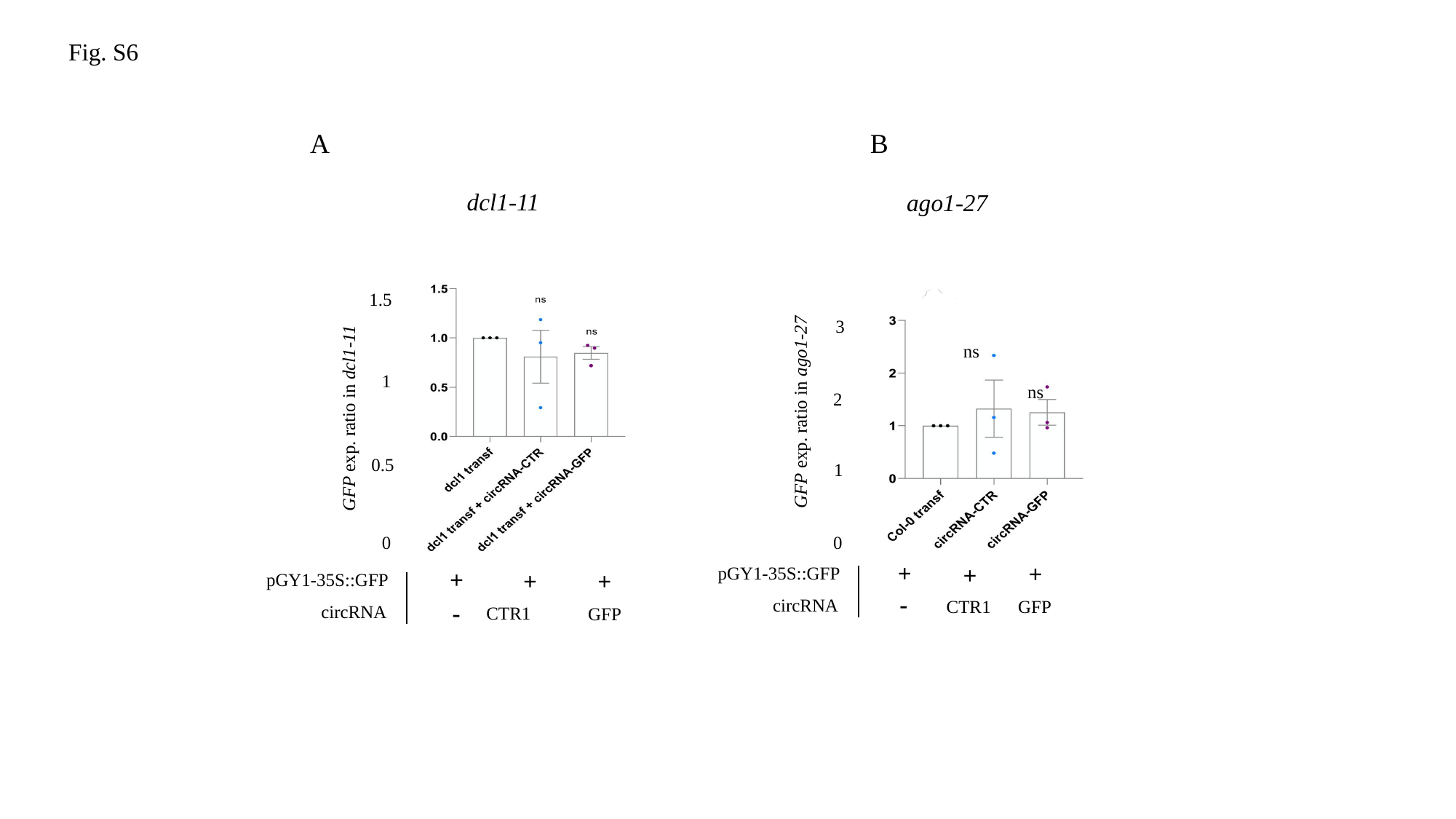

## Slide 12
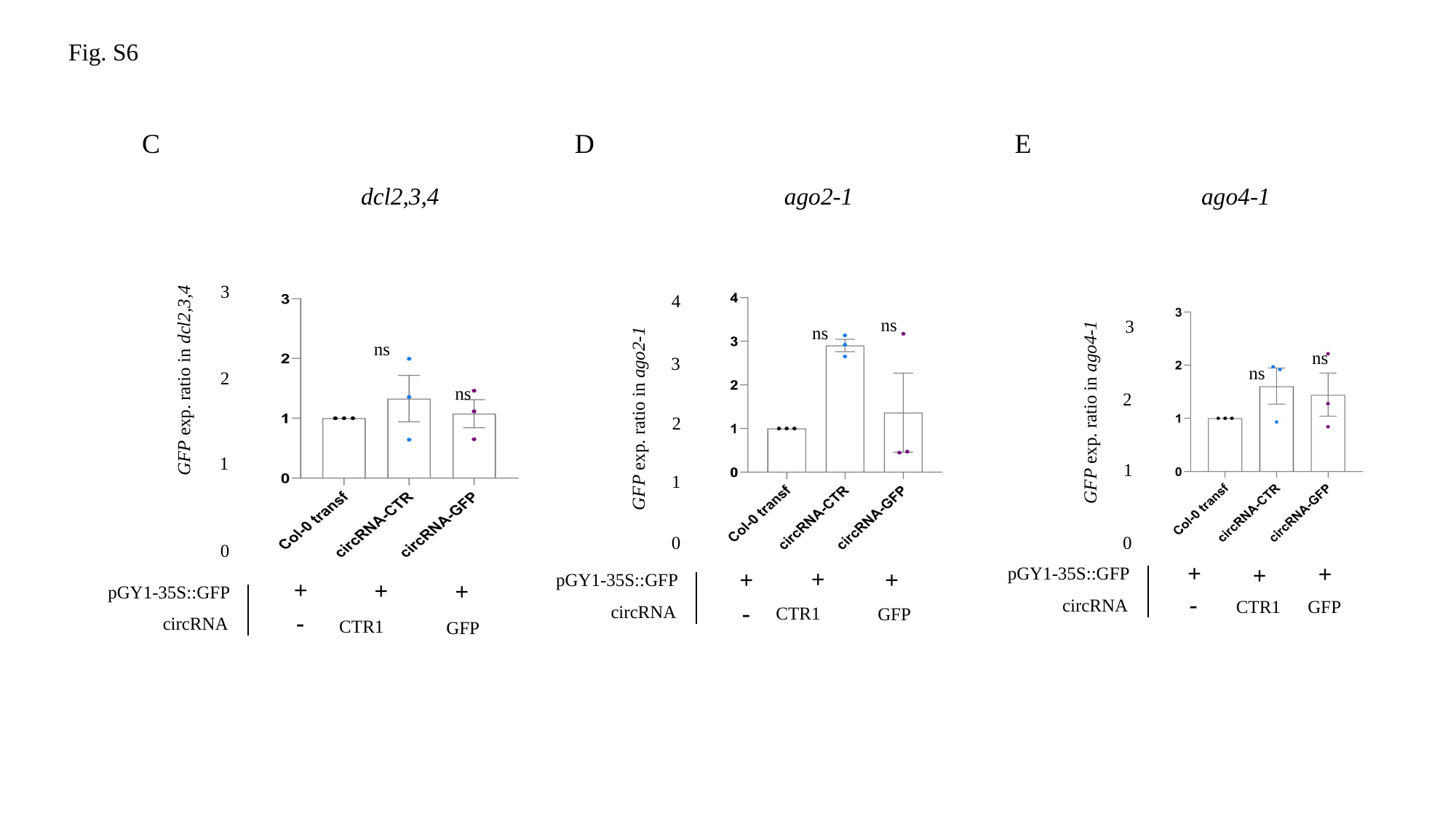

## Slide 13
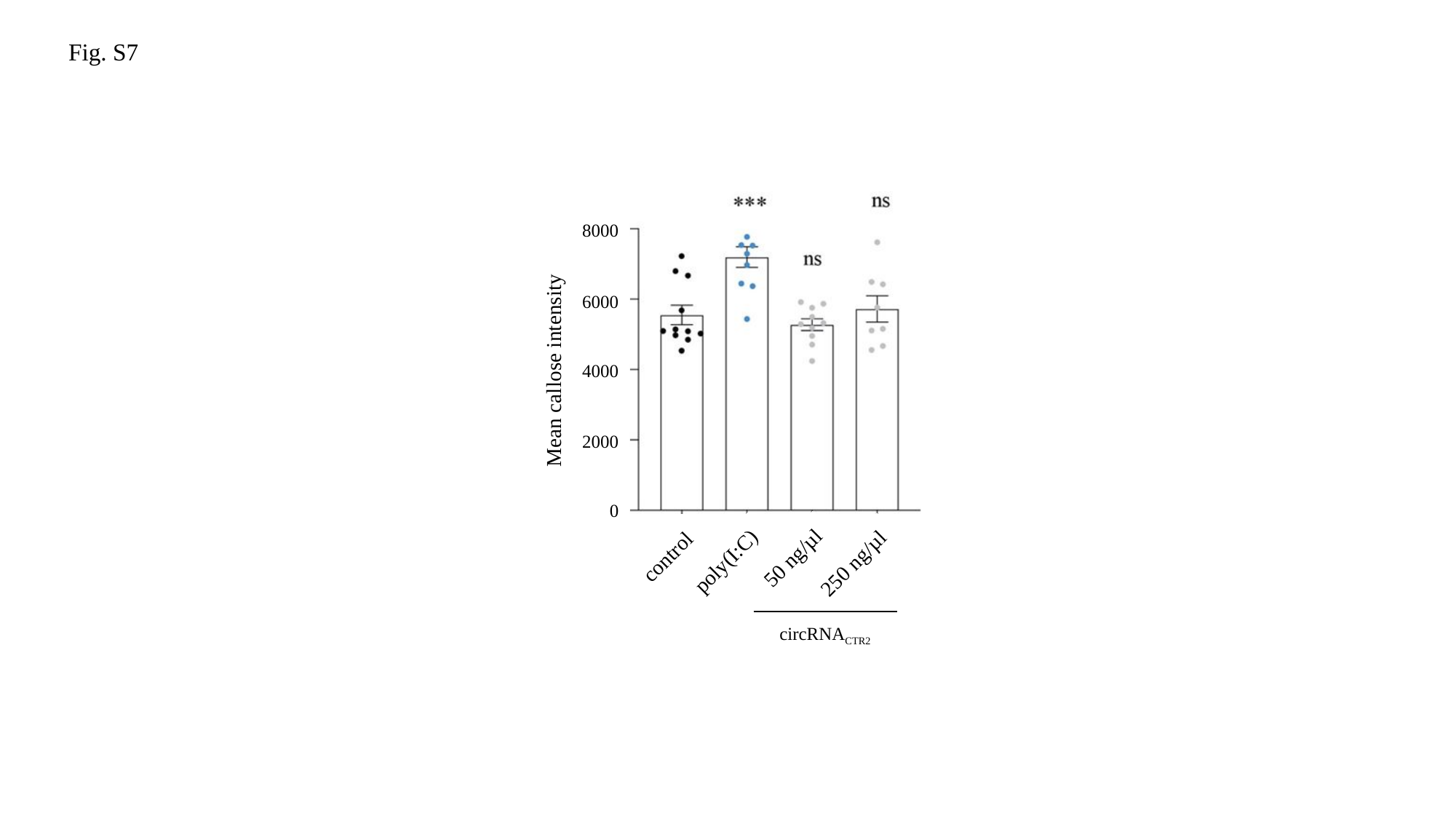

## Slide 14
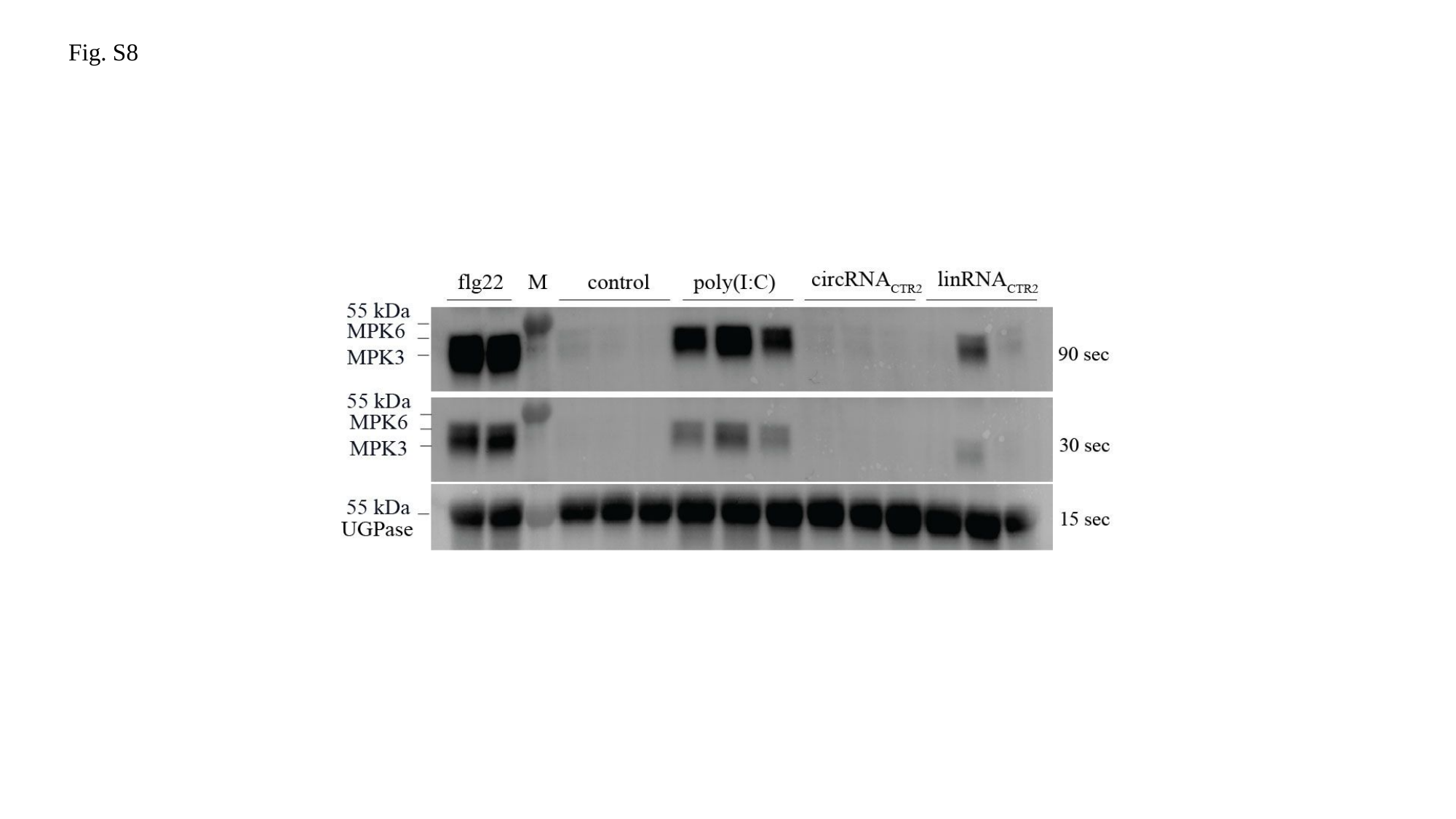

Supplement: Supplementary file 1 — Supplementary file1 (PPTX 70702 KB) [file 299_2025_3512_MOESM1_ESM.pptx]
