# Supplementary material for: Designer circRNAGFP reduces GFP-abundance in Arabidopsis protoplasts in a sequence-specific manner, independent of RNAi pathways
Source: Plant Cell Rep. 2025 May 22;44(6):128. doi: 10.1007/s00299-025-03512-y (PMC12098445; doi:10.1007/s00299-025-03512-y)
Supplement: Supplementary file 2 — Supplementary file2 (DOCX 23 KB) [file 299_2025_3512_MOESM2_ESM.docx]

**Supplementary data and figure legends**

**Figure S1** Topology of designer circRNAs and their target sequences in the *GFP* gene. (A) *GFP*-ORF region nucleotide sequence with color code: the 30 nt target sequence of the 50 nt *GFP* antisense circRNA_GFP_ is highlighted in red. Highlighted in blue is the CaMV35S promotor sequence; highlighted in yellow is the 5’UTR; red font represents the circRNA target sequence (30 nt); start codon ATG underlined in green; stop codon TAA underlined in red; 3’UTR highlighted in green; terminator sequence highlighted in grey. (B) Minimal Free Energy secondary structure: The exact position of the target sequence was selected based on the secondary structure model of the ORF. The red box inside the *GFP* mRNA structure indicates the binding site for the designated antisense circRNA_GFP_. Base pair probability is decoded by different colours. (C) The selection of this region was confirmed by measuring mRNA accessibility using the RNAup software (Vienna RNA Package, http://rna.tbi.univie.ac.at/). The *GFP* mRNA sequence (5'UTR and 3'UTR included) was used with its own antisense RNA sequence to determine RNA accessibility. This analysis served as a guide for selecting sequence sections for antisense circRNA design. Green box indicates higher mRNA accessibility, chosen for the target sequence of the antisense circRNA_GFP_. (D) Sequences and structures of three circRNAs used in this study. The secondary structures of designer circRNAs were determined with the program mfold ([www.unafold.org](http://www.unafold.org/); Zuker 2003). The *GFP* target sequence is colored in green, while randomized control sequences are colored in blue. Respective nt sequences of all circRNA are shown in Table S1.

**Figure S2** Map of plasmids for Arabidopsis protoplast transfection. (A) pGY1-35S::GFP:RFP: The plasmid was prepared by using the pGY1-35S::GFP (Schweizer et al. 1999; see B) plasmid backbone, where RFP was inserted by cloning. (B) pGY1-35S::GFP backbone: The whole construct confers resistance to ampicillin and carbenicillin antibiotics. *GFP* is inserted and its expression is under the control of *Cauliflower Mosaic Virus* 35S (CaMV35S) promoter and terminator. The maps were generated by SnapGene freeware (<https://en.freedownloadmanager.org/Windows-PC/SnapGene-Viewer-FREE.html>.

**Figure S3** Microscopic imaging of GFP fluorescence in Arabidopsis protoplasts. Protoplasts were transfected with 20 µg of plasmid pGY1-35S::GFP and 4 µg of *GFP* antisense circRNA_GFP_, or 4 µg of non-targeting circRNA_CTR1_. (A). After 18 hpt, protoplasts were examined under the microscope using two distinct filters to analyze the ratio of fluorescence levels between the GFP fluorescent protoplasts (λexc 470, λem 525 nm) and red chlorophyll autofluorescence (λ_exc_ 480, λ_em_ 510 nm). Fluorescence intensity was quantified based on images by using ImageJ 1.54p software. The scale bar represents 500 μm. (B). The ratio between green pixels (GFP fluorescence) and red pixels (chlorophyll fluorescence) as calculated with ImageJ represented in the graph. The bar represents the measurements of ≥3 individual pictures taken at various positions. Statistical analysis was performed using one-way ANOVA, where ‘*’ denotes p ≤ 0.05 significant difference between circRNA_GFP_-treated and control protoplasts (transfected without circRNA or circRNA_CTR1_) (Tukey’s test). ns, not significant.

**Figure S4** Microscopic imaging of the dose-dependency of GFP abundance in Arabidopsis protoplasts in response to circRNA treatment. Protoplasts were transfected with 20 µg of plasmid pGY1-35S::GFP and the indicated amount of circRNA_GFP_ (A) or circRNA_CTR1_ (B), respectively. At 18 hpt, protoplasts were inspected under the fluorescence microscope using two different filters to calculate the ratio in fluorescence levels between the GFP fluorescent protoplasts (λ_exc_ 470, λ_em_ 525 nm) and total protoplasts (red chlorophyll autofluorescence, λ_exc_ 480, λ_em_ 510 nm). Fluorescence was measured based on pictures by using ImageJ 1.54p software. The scale bar represents 500 μm.

**Figure S5** Microscopic imaging of the GFP fluorescence in Arabidopsis RNAi mutants. Protoplasts of mutants *dcl1-11* (A,B), *ago1-27* (C,D) and *dcl2,3,4* (E,F) transfected with 20 µg of plasmid pGY1-35S::GFP and 4 µg of non-targeting circRNA_CTR1_ or *GFP* antisense circRNA_GFP_. At 18 hpt, protoplasts were inspected under the fluorescence microscope using two different filters to calculate the ratio in fluorescence levels between the GFP fluorescent protoplasts (λ_exc_ 470, λ_em_ 525 nm) and total protoplasts (red chlorophyll autofluorescence, λ_exc_ 480, λ_em_ 510 nm). Fluorescence was measured based on pictures using ImageJ 1.54p software. The scale bar represents 500 μm. The ratio between green pixels (GFP fluorescence) and red pixels (chlorophyll fluorescence) was calculated with ImageJ for mutants *dcl1-11* (B), *ago1-27* (D) and *dcl2,3,4* (F). Bars represent the average of the measurements of at least 3 pictures taken at different spots with standard error of the mean (SEM). Statistical analysis was performed with one-way ANOVA, where * denotes p≤0.05 and ** denotes p≤0.01 significance vs. control protoplasts (circRNA_CTR1_-transfected or nontreated (mock) protoplasts (Tukey's test). ns, not significant.

**Figure S6** RT-qPCR analysis of the amount of *GFP* transcripts in Arabidopsis protoplasts from RNAi mutants (*dcl1-11*, *ago1-27*, *dcl2,3,4*, *ago2-1*, *ago4-1*) upon treatment with circRNA. Protoplasts were transfected with 20 µg of pGY1-35S::GFP plasmid and 4 µg of circRNA_GFP_ or non-targeting circRNA_CTR1,_ respectively. Relative *GFP* expression was measured after 18 hpt. Values were normalized to the housekeeping gene *Ubiquitin*. Bars represent an average of three independent biological experiments pooled together with standard error of the mean (SEM). No statistically significant differences between treatments and genotypes were detected (one sample *t*-test for (A), (B), (C), and (E) and one sample Wilcoxon test for (D), p≥ 0.05). ns, not significant.

**Figure S7** Mean values of callose intensity levels at PD in individual images taken of epidermal cells of leaf disks treated with a 0.1% aniline blue solution containing water (control), 500 ng/µL of poly(I:C), or either 50 ng/µL or 250 ng/µL of circRNA_CTR2_. Error bars show the standard error of the mean. Parametric mean value variances were tested by one-way ANOVA followed by Dunnett’s multiple comparisons test. ***, p$\leq$ 0.001; ns = non-significant.

**Figure S8** Immunoblots to detect mitogen-activated protein kinase (MAPK) phosphorylation in Arabidopsis leaves probed with antibodies against phosphor-p44/42 ERK. Leaf disks were vacuum infiltrated with either water (negative control), 1 µM flg22, 1500 ng/µl poly(I:C) ($\sim$3 µM) or 50 ng/µl ($\sim$3 µM) of each linRNA_CTR2_ or circRNA_CTR2_. Equal protein loading was verified using an anti-UGPase antibody. The blot image is a composite of the membrane with PageRuler^TM^ pre-stained protein ladder (M) and after 90 sec or 30 sec exposures.
