# Supplementary material for: Designer circRNAGFP reduces GFP-abundance in Arabidopsis protoplasts in a sequence-specific manner, independent of RNAi pathways
Source: Plant Cell Rep. 2025 May 22;44(6):128. doi: 10.1007/s00299-025-03512-y (PMC12098445; doi:10.1007/s00299-025-03512-y)
Supplement: Supplementary file 3 — Supplementary file3 (DOCX 30 KB) [file 299_2025_3512_MOESM3_ESM.docx]

**Table S1:** Nucleotide sequences of circRNAs and linRNAs used in this study.

| Name | Sequence (5’-3’) |
| --- | --- |
| ^1^circRNA_GFP_ | GGGAGUAAGCUCGUGCUGCUUCAUGUGGUCGGGGUAGCGGGCUUACAGUA |
| ^1^circRNA_CTR1_ | GGGAGUAAGCAGAUGCGCACCGCACAGAUGCGCACGCUUACAGUA |
| ^1^circRNA_CTR2_ | GGGAGUAAGCAAAAGUCAGUGAGUCAGUGUAAUACGGGAGGAUACCCGCUGUCAAAGCUUACAGUA |

^1^ This sequence is identical to the corresponding linRNA sequence. Underscored nt are needed for circularization.

**Table S2:** List of oligonucleotides used in this study.

| Primer Name | Sequence of primers/oligonucleotides | Application |
| --- | --- | --- |
| *GFP*_F | GACGTAAACGGCCACAAGTTC | qPCR |
| *GFP*_R | AAGTCGTGCTGCTTCATGTG | qPCR |
| *At* *Ubiquitin*_F | GCTTGGAGTCCTGCTTGGACG | qPCR |
| *At* *Ubiquitin*_R | CGCAGTTAAGAG GACTGTCCGGC | qPCR |
| *AtEF1-α_F* | CTGTTGTAACAAGATGGATGCC | qPCR |
| *AtEF1-α_R* | CCCTCGAATCCAGAGATTGG | qPCR |
| *RFP*_F | GCGAGATCAAGATGAGGCTGA | qPCR |
| *RFP*_R | TAGTCCTCGTTGTGGGAGGT | qPCR |
| circRNA_GFP__F | TAATACGACTCACTATAGGGAGTAAGCTCGTGCTGCTTCATGTGGTCGGGGTAGCGGGCTTACAGTA | circRNA production |
| circRNA_GFP__R | TACTGTAAGCCCGCTACCCCGACCACATGAAGCAGCACGAGCTTACTCCCTATAGTGAGTCGTATTA | circRNA production |
| circRNA_CTR1__F | TAATACGACTCACTATAGGGAGTAAGCAGATGCGCACCGCACAGATGCGCACGCTTACAGTA | circRNA production |
| circRNA_CTR1__R | TACTGTAAGCGTGCGCATCTGTGCGGTGCGCATCTGCTTACTCCCTATAGTGAGTCGTATTA | circRNA production |
| circRNA_CTR2__F | TAATACGACTCACTATAGGGAGTAAGCAAAAGTCAGTGAGTCAGTGTAATACGGGAGGATACCCGCTGTCAAAGCTTACAGTA | circRNA production |
| circRNA_CTR2__R | TACTGTAAGCTTTGACAGCGGGTATCCTCCCGTATTACACTGACTCACTGACTTTTGCTTACTCCCTATAGTGAGTCGTATTA | circRNA production |
